# Charged Polymers Transport under Applied Electric Fields in Periodic Channels

**DOI:** 10.3390/ma6073007

**Published:** 2013-07-19

**Authors:** Sorin Nedelcu, Jens-Uwe Sommer

**Affiliations:** 1 Leibniz-Institut für Polymerforschung Dresden, Hohe Str. 6, Dresden 01069, Germany; 2 Institute of Theoretical Physics, Technische Universität Dresden, Dresden 01069, Germany; E-Mail: sommer@ipfdd.de

**Keywords:** molecular dynamics, polyelectrolytes, confinement

## Abstract

By molecular dynamics simulations, we investigated the transport of charged polymers in applied electric fields in confining environments, which were straight cylinders of uniform or non-uniform diameter. In the simulations, the solvent was modeled explicitly and, also, the counterions and coions of added salt. The electrophoretic velocities of charged chains in relation to electrolyte friction, hydrodynamic effects due to the solvent, and surface friction were calculated. We found that the velocities were higher if counterions were moved away from the polymeric domain, which led to a decrease in hydrodynamic friction. The topology of the surface played a key role in retarding the motion of the polyelectrolyte and, even more so, in the presence of transverse electric fields. The present study showed that a possible way of improving separation resolution is by controlling the motion of counterions or electrolyte friction effects.

## 1. Introduction

Experiments and computer simulations show that charged chains in free solution under an external applied electric field migrate with the same electrophoretic velocity independent of the chain size [1] (except for very small rod-like polyions). To further derive an analytical expression for the electrophoretic velocity, the charge density within the polymer domain must be calculated and, also, the hydrodynamic friction due to the solvent [2]. Typically, two interesting limits are considered. First, in the limit of a completely permeable coil to the solvent molecules, which flow unperturbed through the polymer coil, the fluid resistance is proportional to the degree of polymerization, or chain length *N*. In the second limit, the polymer coil is viewed as an impenetrable sphere (of radius $R_{s}$), with a very large local segment density. The fluid resistance is that of Stokes’ law, which means that friction is proportional to chain size [3] and scales linearly with $R_{s}$.

For the electrophoretic velocity *v*, in the limit of small electric potentials and neglecting relaxation effects (the counterions surrounding the charged polymer are not subject to convection), Hermans and Fujita [4,5] obtained an analytical expression as a result of the porous sphere model, which included the dependency on the electric field *E*, the radius of the porous sphere $R_{s}$, the internal segment density $\nu_{m}=3N/4\pi R_{s}^{3}$, the hydrodynamic shielding parameter $K_{H}^{2}=2\nu_{m}/3\eta$, the fluid viscosity *η*, the Debye length $\kappa^{-1}$, and the fixed charged density of the polyion $\rho_{f}$. It was assumed that the fluid velocity at an arbitrary position generated by applied force at the origin could be replaced in the Oseen relation by its average value over a spherical surface. We remark here that in strong confinements this cannot be strictly applicable, since there, the fluid flow velocity is highly non-isotropic. In the particular limit of completely permeable coils (*i.e.*, $K_{H}R_{s}\to 0$) the electrophoretic velocity is $v=\rho_{f}E/\nu_{m}\gamma$, where *γ* is the friction coefficient. This means that the mobility of a polyion is equal to the mobility of a segment. The other limit of a macromolecule behaving as a compact sphere (*i.e.*, $K_{H}R_{s}\to \infty$) leads to the following expression, $v=2\rho_{f}R_{s}^{2}e^{-p}/\left(3\eta p^{2}\right)\Gamma \left(p\right)coshp$, where $p=\kappa R_{s}$ and $\Gamma \left(p\right)=p^{2}/\left(3+p^{2}\right)$, in agreement with Henry’s theory [6]. If relaxation effects are also taken into account, then, an expression for the electrophoretic velocity, which is dependent on the electric conductivities inside and, respectively, outside the polymeric domain, has been obtained by Longworth and Hermans [7]. The inclusion of relaxation effects makes the electrophoretic velocity dependent upon the degree of polymerization, but the effect is thought to be very small.

Furthermore, Katchalsky *et al.* [8] worked out an analytical expression for the friction coefficient of the translational motion of the polymer, if the chain conformation is not Gaussian. The friction has quite a complicated dependence on the chain length, and it is not only the sum of segmental frictions, as for the case of freely-drained polymers. On the other hand, as we mentioned in the beginning, experimental results show that the mobility of the chains μ=v/E, or $\mu =Q_{eff}/\xi$, if we denote by $Q_{eff}$ the effective charge of the polyion, does not depend on chain length *N*. Two of the most restrictive conditions were the averaging of the hydrodynamic interactions, which works well for near equilibrium transport, and the use of linearized Poisson-Boltzmann equation, if the polyions are weakly charged.

A modern review of these effects of electroosmosis and counterion penetration on electrophoresis of positively charged spherical permeable particles has been recently presented by Bhattacharyya and Gopmandal [9]. Using numerical techniques, the authors showed good agreement with previous theories, due to Hermans and Fujita and, more recent, results by Keh and Chen [10]. The electrical force and the hydrodynamic drag along the flow direction has been calculated by integrating on the surface of the particle the Maxwell stress tensor and hydrodynamic stress tensor. While this approach has been applied to spherical particles, its extension to arbitrary particle shapes, as for the case of confinement, is an attractive alternative to full molecular dynamics simulations.

Another recent review on DNA molecules in confinement is presented in [11]. Experiments on electrophoresis of DNA molecules in artificial nano-channel matrices are reported by Wang *et al.* [12]. The authors find that the mobility of the strongly confined DNA depends on the degree of confinement. At strong confinement, it decreases with chain length, as is the case of gel electrophoresis, and increases at weaker confinement. Conformation dependence of DNA electrophoretic mobility in a converging channel is shown by Liao *et al.* [13]. Similar findings are presented in [14], including the case of pulse applied electric fields or numerical investigations in [15].

From these works, it is clear that the balance between the electrostatic forces, the hydrodynamic drag due to the solvent and the induced pressure field determine the motion of the polyions. It is our aim to investigate these effects in more detail and, in particular, for the case of confinement. Here, the macroscopically observed linear relationship between friction force and contact area can be extended to the nanoscale, with the contact area being proportional to the number of interacting atoms across the contact. There are, however, interesting effects related to surface friction, as we shall see below, if surface roughness forbids the stretching of the chains. Related experimental work showing a reduction of fluid friction atop undulating surfaces is presented, for example, by Vlachogiannis and Hanratty [16] and more recent mesoscopic simulations of pressure-driven fluid flow in periodically grooved microchannels by Kasiteropoulou [17].

**Figure 1:**
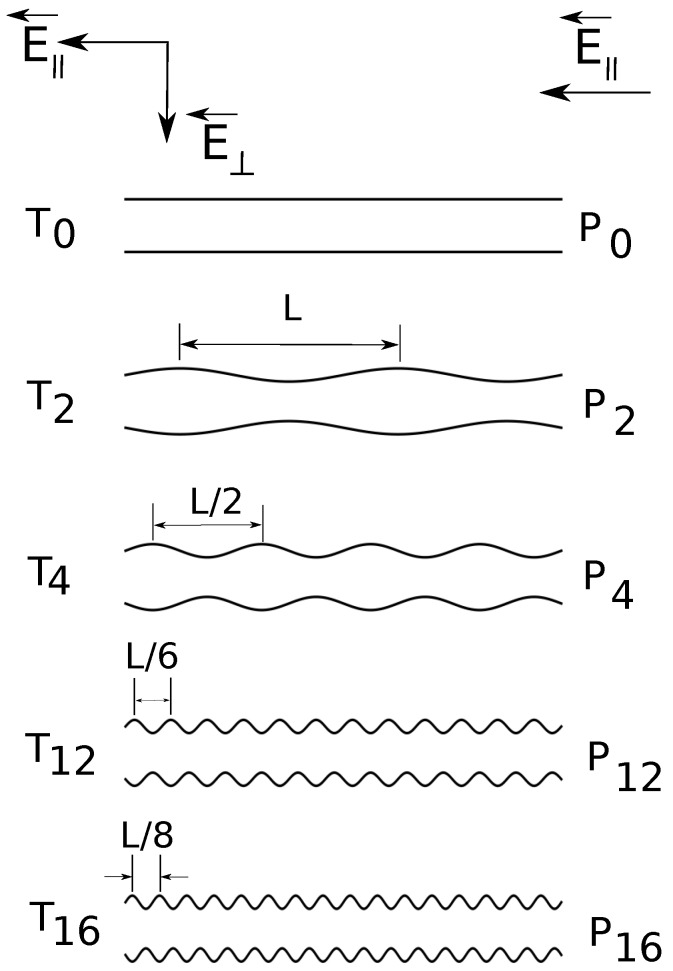
Cross-section schematic illustration (from top to bottom) of constant radius (6σ) and variable diameter straight cylinders of increasing surface undulations. The depth of surface undulations is constant, 25% of the cylinder radius. $P_{0}$ denotes the combination of straight cylinders and parallel applied electric fields ${\vec{E}}_{∥}$, $T_{0}$ denotes the combination of straight cylinders and both parallel and transverse applied fields ${\vec{E}}_{\left|\right|}+{\vec{E}}_{⊥}$, *etc*.

In the present simulations, the shape of the confinement is either a straight cylinder or a cylinder with periodically varying diameter. The applied field can have: a longitudinal component ${\vec{E}}_{∥}$, in the axial direction only or both a longitudinal and a transverse component ${\vec{E}}_{∥}+{\vec{E}}_{⊥}$. In Figure 1, we present a schematic representation of the geometries. We briefly answer here the question of why do we need such a setup? First of all, the component ${\vec{E}}_{∥}$ is the driving field, which has the same role as a pressure gradient [18]. Second, the transverse component ${\vec{E}}_{⊥}$ enforces a separation of electroosmotic flows of counter ions and ions of added salt. We showed earlier that such separation improves resolution [19]. In the present study, we go a step further and estimate quantitatively electrolyte friction coefficients and fluid flow velocities inside the charged polymers. The surface undulations are introduced in order to understand the interrelation between geometry shape and electrolyte friction.

## 2. Method

The charged polymers are bead-spring models consisting of *N* beads, each carrying a unit negative electric charge and connected by a finitely extensible nonlinear elastic (FENE) potential, $U_{FENE}=-\left(1/2\right)kR_{0}^{2}log\left(1-{\left(r/R_{0}\right)}^{2}\right)$, with spring constant $k=7k_{B}T/\sigma^{2}$, and maximum extension, $R_{0}=2.4\sigma$, where *σ* is the unit length and $k_{B}T$ is the unit energy. To simulate salt conditions, we added monovalent counterions with a concentration of $0.0291\sigma^{-3}$ and also coions, such that the system is electrically neutral. All charged monomers interact with each other through Coulomb interactions, $U_{C}=k_{B}Tl_{B}/r$, where we set $l_{B}=1\sigma$, which is the Bjerrum length of the solution. The distance between charged groups on the polyelectrolytes is b≈0.93σ, which means that the charge density is $\xi =l_{B}/b\approx 1$ and condensation of counterions does not occur [20]. All the particles (polymer or fluid monomers, counterions, coions) are modeled as spheres that interact by purely repulsive Lennard-Jones (LJ) potential, $U_{LJ}=4\epsilon_{LJ}\left({\left(\sigma /r\right)}^{12}-{\left(\sigma /r\right)}^{6}\right)$, which is cut at ${\left(2\sigma \right)}^{1/6}$ and shifted to zero. Here, we choose $\epsilon_{LJ}=1k_{B}T$. The walls of the confinement are made of uncharged monomers, which are assigned fixed positions in space. The length of the simulation box is much larger than the contour length of the charged chain. For example, we choose a minimum cylinder length of 565σ and a maximum of approximately 2×565σ for the longest chain. In all cases, the number density of counterions (and coions) was changed accordingly to the set value of $0.0291\sigma^{-3}$. Similarly, for any length of the simulation box, the number density of fluid monomers was fixed at $0.82\sigma^{-3}$. Periodic boundary conditions are imposed in one direction only, along the cylinder axis. The fluid monomers are explicitly modeled as spherical particles and interact with all the other particles through the Lennard-Jones potential. We choose the same radius for all particles. The number density of fluid monomers is set to $0.82\sigma^{-3}$. We use a Nosé-Hoover thermostat. In all studied cases, the applied longitudinal electric field is $E_{∥}=1$ and the transversal field is $E_{⊥}=1$, in units of $k_{B}T/\left(e\sigma \right)$. We note here that for the chosen concentration of free charges, the Debye length is $\lambda_{D}=1/\sqrt{4\pi \times l_{B}\times 2\times 0.0291}=1.17\sigma$. This characteristic length scale of the system becomes less defined for the case of transverse fields where there is a separation of flows of counterions and coions of salt and the system is not homogenous. The time step used in the simulations is τ=0.008 in units of $\sigma \sqrt{m/k_{B}T}$, and the mass of each particle is unity. All physical quantities are, therefore, given in reduced Lennard-Jones units.

The radius of the uniform straight cylinder is 6σ, and the depth of surface undulations is 25% of the cylinder radius; the minimum cylinder radius is 4.5σ and the maximum is 7.5σ (Figure 1). The surface undulations are sinusoidal waves of fundamental frequency $\omega_{0}=2\pi /L$, where L=57.11σ. Geometries $P_{2}$ and $T_{2}$ have frequency $\omega_{2}=\omega_{0}$; $P_{4}$ and $T_{4}$ have frequency $\omega_{4}=2\omega_{0}$; $P_{12}$ and $T_{12}$ have frequency $\omega_{12}=6\omega_{0}$; $P_{16}$ and $T_{16}$ have frequency $\omega_{16}=8\omega_{0}$.

The hydrodynamic friction force, ${\vec{F}}_{H}$, experienced by a charged polymer monomer is proportional to the difference between the velocity of the bead, $\vec{v}$, and the velocity of the fluid, ${\vec{v}}_{f}$, at the location of the bead, ${\vec{F}}_{H}=-\xi \left(\vec{v}-{\vec{v}}_{f}\right)$. The beads of the polyion are hydrodynamically interacting with each other, and the flow at the position of, say, bead *i* include perturbations due to the other beads, counterions and coions of added salt. In our simulations, the solvent is explicit, and therefore, the fluid velocity is obtained by averaging the instantaneous velocities of solvent monomers around each bead, *i*. The friction forces are, thus, position- and configuration-dependent.

**Figure 2:**
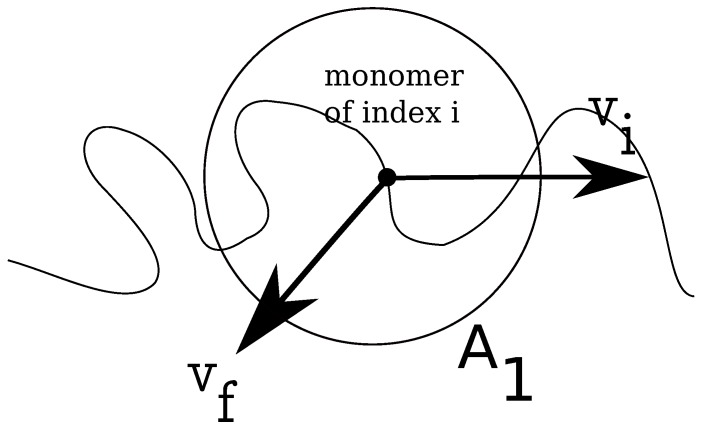
Schematic illustration of fluid control volume, $A_{1}$, around an arbitrary monomer of a charged chain, which has instantaneous velocity ${\vec{v}}_{i}$. The local fluid velocity at the position of monomer *i* is noted ${\vec{v}}_{f}$.

**Figure 3:**
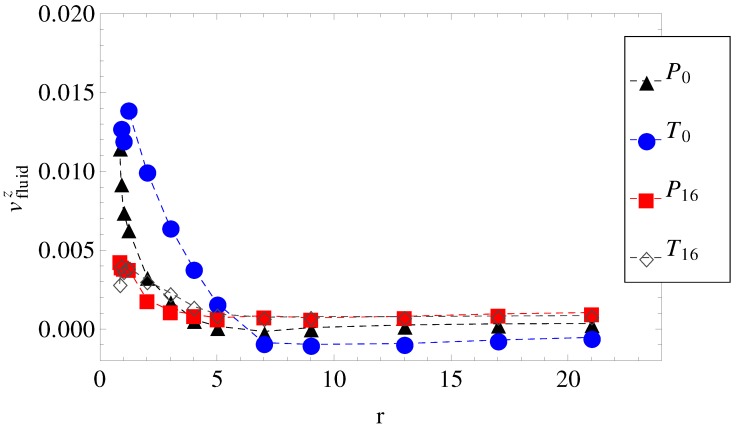
The axial component of the fluid velocity around the polyion as a function of radial distance *r*, for N=180. The legend notation is the same as in Figure 1.

In Figure 2, we show how the local fluid velocity around an arbitrary monomer of the charged chain is computed. The calculation proceeds as follows: we index all monomers of the charged chain from one to *N*. We set, then, a distance *r*, which is the radius of a spherical domain $A_{1}$, around each monomer, i=1,N. After this, we make a list of all fluid monomers contained in all volumes $A_{1}$, which were drawn around each monomer, i=1,N, within distance *r*, from the polyelectrolyte. In this list, some fluid monomers may appear twice, because the spherical volumes $A_{1}$ overlap. We identify, then, all unique fluid monomers contained in all volumes $A_{1}$, of distance *r* from the polyelectrolyte. Since the velocities of all fluid monomers are known, we compute the time averages for distances between *r* and r+δr, where δr is a small parameter of the order of *σ*. The final result for a chain with N=180 monomers, for some geometries, is shown in Figure 3. The fluid velocities are in the reference frame of the laboratory and projected on the z-axis (the direction of the longitudinal field). As expected, at large distances from the polyion the relative velocities are close to zero, while the largest values are in the immediate vicinity of the charged polymer, where the fluid is mostly perturbed. The averages are taken every 1000 time steps, while the total number of time steps for each chain length and each geometry is about $10^{7}$ steps.

By plotting, for example, the average fluid velocity, $v_{f}=v_{f}\left(r\right)$, and the number of counterions contained in a spherical shell of radius *r*, as a function of distance *r* from the center of the macroion, Chatterji and Horbach [21] were able to estimate the role of effective charges in the electrophoresis of highly charged colloids.

With respect to the role played by electrolyte friction effects, which are retardation effects due to the counterions flowing in the opposite direction to the polyelectrolyte and slow coions flowing alongside the charged polymer, we estimate these retardation forces, of electric nature, from Schurr’s expression for the static friction of a colloidal macroion [22]. Assuming that the fixed point charge on a bead of the polymer is $q_{i}$, each of these charges interact with each other and with the free ions through Coulomb forces, ${\vec{F}}_{C}\left({\vec{r}}_{i},t\right)=q_{i}/\left(4\pi \epsilon_{0}\epsilon \right)\sum_{j=1}^{n}(q_{j}/|{\vec{r}}_{ij}{|}^{2}){\vec{r}}_{ij}$, where $q_{j}$ are point charges representing monovalent counterions and salt ions. The distance between $q_{i}$ and $q_{j}$ is noted with ${\vec{r}}_{ij}={\vec{r}}_{i}-{\vec{r}}_{j}$; $\epsilon_{0}$ and *ϵ* are the permittivity of the free space and, respectively, the relative permittivity of the solvent. In Schurr’s theory, it is assumed that the fluctuating force on the polyion due to the small ions is completely uncorrelated with that due to the solvent, and therefore, the fluctuating ionic force contributes an independent additive contribution on top of the Stokes friction. The electrolyte friction is obtained from the time integral of the auto-correlation function of Coulomb forces:

(1) $$\xi_{C}=1/\left(k_{B}T\right)\int_{0}^{\infty }\langle {\vec{F}}_{C}\left(0\right)\cdot {\vec{F}}_{C}\left(t\right)\rangle dt$$

Such theoretical developments are quite powerful and show an excellent good agreement with experiments. Moreover, Schurr’s theory is parameter-free. The detailed calculation of electrolyte friction in Equation (1) is as follows: at an arbitrary time, *t*, for each charged polymer monomer, i=1,N, we choose an arbitrary distance, *r*, and draw a control volume, $A_{1}$, around each monomer of the charged chain, similar to the problem of estimating the local fluid velocity (Figure 2). Further, we calculate the relative distances between the free ions contained in the respective volume $A_{1}$, and the monomer *i*, which allows us to estimate the Coulomb forces ${\vec{F}}_{C}\left({\vec{r}}_{i},t\right)$ on each monomer i=1,N, where ${\vec{r}}_{i}$ is the coordinate of monomer *i*. We define monomeric friction coefficients from the time integral of the auto-correlation function of Coulomb forces as follows:

(2) $$\xi_{C}^{i}=\left(1/k_{B}T\right)\int_{0}^{\infty }\langle \left({\vec{F}}_{C}\left({\vec{r}}_{i},t\right)-{\vec{F}}_{C}^{av}\left(t\right)\right)\cdot \left({\vec{F}}_{C}\left({\vec{r}}_{i},0\right)-{\vec{F}}_{C}^{av}\left(0\right)\right)\rangle dt$$

where ${\vec{F}}_{C}^{av}=\left(1/N\right)\sum_{i=1}^{N}{\vec{F}}_{C}\left({\vec{r}}_{i},t\right)$ is the average Coulomb force exerted on the polyelectrolyte at time, *t*, by the free ions. The total electrolyte friction is the average of individual coefficients:

(3) $$\xi_{C}=\frac{1}{N}\sum_{i=1}^{N}\xi_{C}^{i}$$

**Figure 4:**
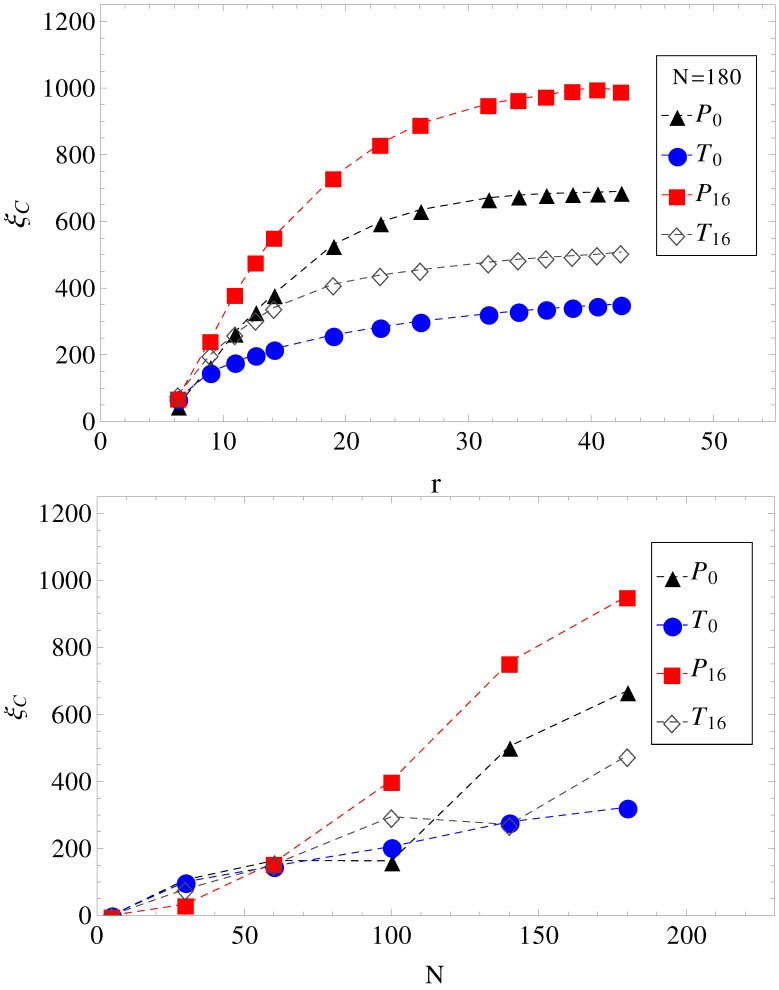
(Top) Electrolyte friction $\xi_{C}$ as a function of distance *r* from the polyelectrolyte (N=180) for limiting cases of smooth and, respectively, wavy surfaces, in parallel (geometries $P_{0}$, $P_{16}$) and in parallel with perpendicular applied fields (geometries $T_{0}$, $T_{16}$). (Bottom) Plateau values (large *r*) of electrolyte friction $\xi_{C}$ as a function of chain length *N* for the same geometries, $P_{0}$, $T_{0}$, $P_{16}$ and $T_{16}$.

In Figure 4, we show the electrolyte friction as a function of distance *r*, for N=180, and the plateau values $\xi_{C}$, at large *r* for the other chain lengths. We apply the same formalism, also, when a transverse electric field is superimposed on the driving longitudinal electric field. We remark here that the term under the integral in Equation (2) has a fast exponential decay in time, but also presents a long time tail that decays as $t^{-3/2}$, which is an indication of hydrodynamic memory effects [23].

## 3. Results and Discussion

**Figure 5:**
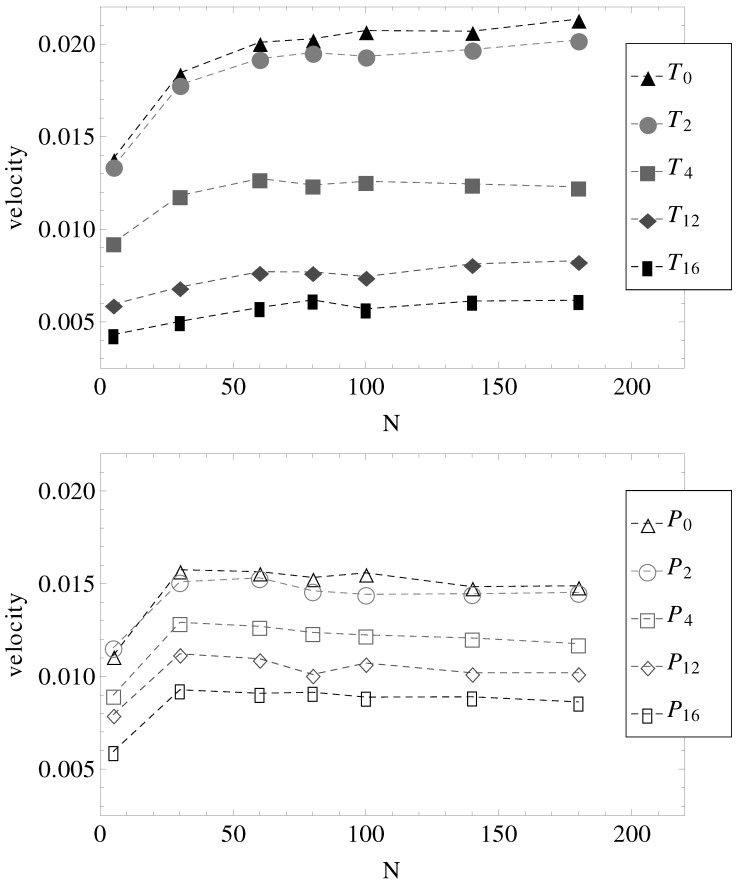
Electrophoretic velocities of charged chains of length *N* in uniform (index “0”) and variable diameter straight cylinders (indices “2”, “4”, “12” and “16” are in the increasing order of the number of undulations per wavelength, as shown in Figure 1). (Top) The constant applied electric field has both longitudinal and transverse components. (Bottom) The constant applied electric field is parallel to the symmetry axis.

Here, we consider equal longitudinal and transverse electric fields, *i.e.*, $|{\vec{E}}_{∥}\left|=\right|{\vec{E}}_{⊥}|=1$ in LJ units and fixed ionic strength of the buffer solution. Only the length *N* of the charged chains and the number of undulations per wavelength are varied (Figure 1). The measured electrophoretic velocities of the charged chains are presented in Figure 5. Two observations are most important.

First, in parallel fields (geometries $P_{0}$–$P_{16}$), all chains with N>30 have more or less the same electrophoretic velocity. It appears, therefore, that by introducing surface undulations, the electrophoretic velocities decrease in value, but remain independent of *N*. It can be straightforwardly assumed that in these cases, the hydrodynamic, electrolyte and surface friction forces all scale with the same power of *N*.

The second observation concerns the effect of the transverse electric field. It appears that in nearly smooth cylinders with both longitudinal and transverse electric fields (geometries $T_{0}$ and $T_{2}$), there is a slight increase in electrophoretic velocities, at least up to chain lengths N<100. Perhaps more remarkable, in geometries $T_{0}$ and $T_{2}$, the electrophoretic velocities are higher than in geometries $P_{0}$ and $P_{2}$, while in geometries $T_{12}$ and $T_{16}$, the velocities are lower than in geometries $P_{12}$ and $P_{16}$ (Figure 5).

**Figure 6:**
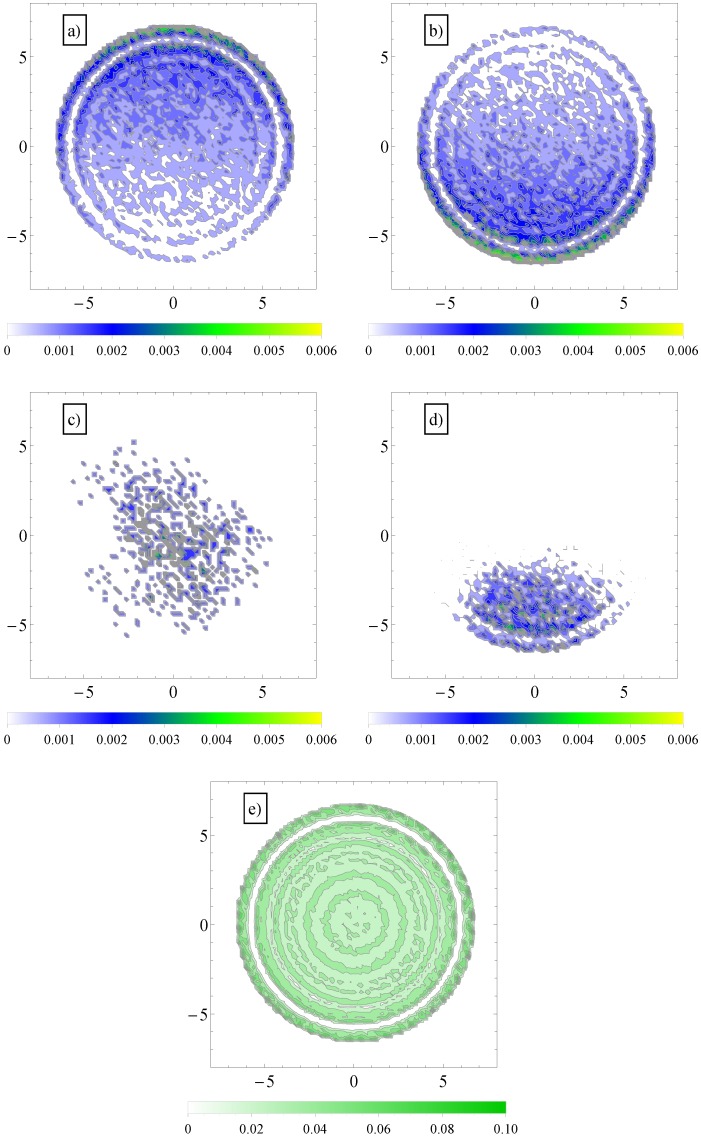
(**a**) Contour plot of average number density distribution of counterions and (**b**) coions of added salt in a cross-section perpendicular to the axis of the cylinder geometry, $T_{12}$. The applied field has both a longitudinal, ${\vec{E}}_{∥}$, and a transversal component, ${\vec{E}}_{⊥}$; (**c**) Similar contour plots of average number density distribution of polymer chain monomers (in the largest section area) in longitudinal fields and (**d**) in both longitudinal and transversal applied fields; (**e**) Similar distribution of fluid monomers in geometry, $T_{12}$. In the vicinity of the walls, the monomers arrange themselves in layers.

To explain this effect, we note first that in transverse fields, there is a separation of electro-osmotic flows of counterions and coions of salt. We show these two distributions in Figure 6.

This separation effectively creates two fluid streams, each flowing in opposite direction to each other. The polyelectrolytes swim in the fluid stream created by the coions of added salt. Because it is mostly surrounded by coions, which flow in the same direction, there are less counterions heading in the polyelectrolyte way, and therefore, there is less retardation from counterions. The conclusion is that a transverse field always makes polyelectrolytes move faster. This can be seen, for example, in Figure 4. The electrolyte friction in $T_{0}$ is smaller than in $P_{0}$. Similarly, the electrolyte friction in $T_{16}$ is smaller than in $P_{16}$. This means that electrolyte friction effects alone predict the inequalities, $v_{T_{0}}>v_{P_{0}}$ and $v_{T_{16}}>v_{P_{16}}$. However, this is not true. Only $v_{T_{0}}>v_{P_{0}}$ is true, and $v_{T_{16}}>v_{P_{16}}$ is false.

**Figure 7:**
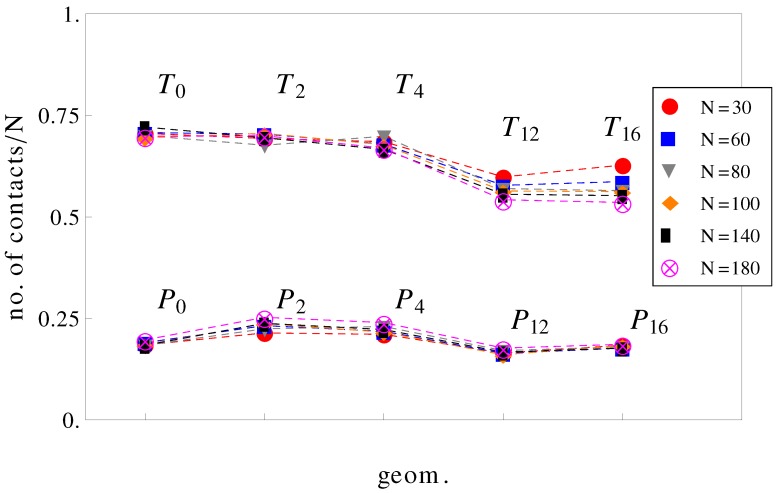
Average number of contacts with the walls, normalized by chain length *N* as a function of geometry (see Figure 1). The top curve is for applied fields with both longitudinal and transversal components, and the bottom curve is for fields parallel to the symmetry axis.

**Figure 8:**
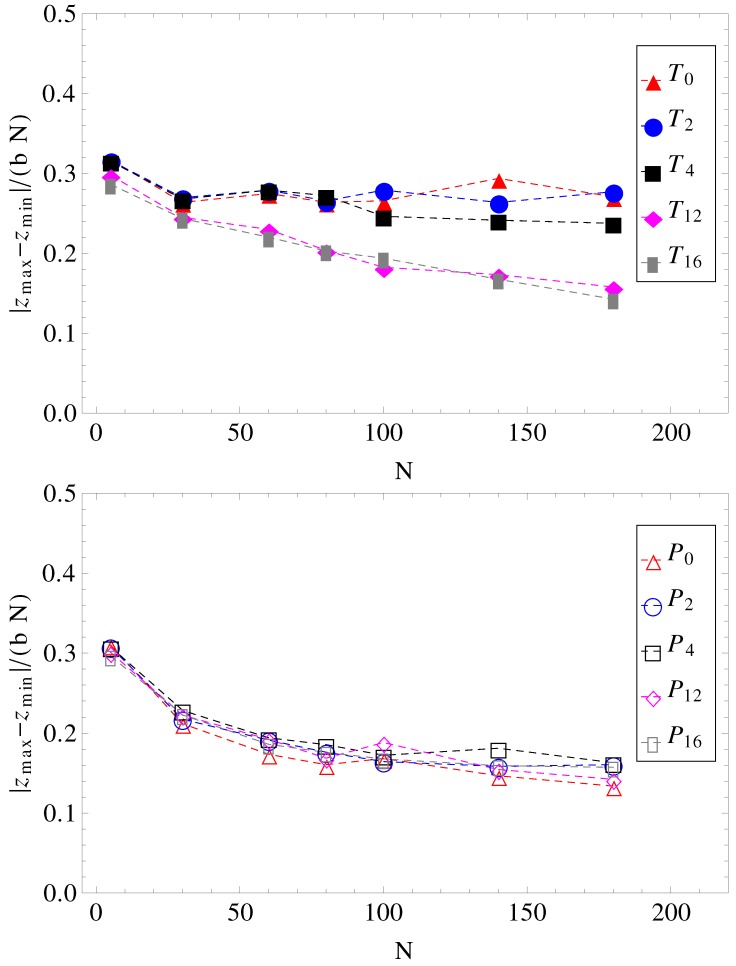
Projections of charged chain extension on the z-axis, normalized by average bond length *b*, and chain length *N*, as a function of chain size (for legend, see Figure 1).

It can be further assumed that in geometries with a high number of surface undulations, the surface friction plays a predominant role in retarding the motion of the charged chains. In this respect, we calculated the average number of contacts between polyions monomers and the walls of the confinement (Figure 7). As expected, the number of contacts is higher in cases where the perpendicular field pushes the charged chains onto the walls of the confinement than in cases where there is no transverse field. This means that surface friction effects alone would give the inequalities, $v_{T_{12}}<v_{P_{12}}$ and $v_{T_{16}}<v_{P_{16}}$. While this is true, surface friction cannot explain why $v_{T_{0}}>v_{P_{0}}$. It may also appear unexpected that the number of contacts with the walls decreases when the number of undulations increases (compare, for example, geometries $T_{0}$ and $T_{16}$ in Figure 7). In these situations ($T_{12}$, $T_{16}$), the chains shrink compared to geometries $T_{0}$ and $T_{2}$ and cannot stretch in the direction of motion. The number of contacts is therefore reduced. The average relative extensions $|z_{max}-z_{min}|$ of the chains in the *z*-direction, where $z_{max}$ and $z_{min}$ are the *z*-coordinates of the leftmost and rightmost monomers, are shown in Figure 8.

**Figure 9:**
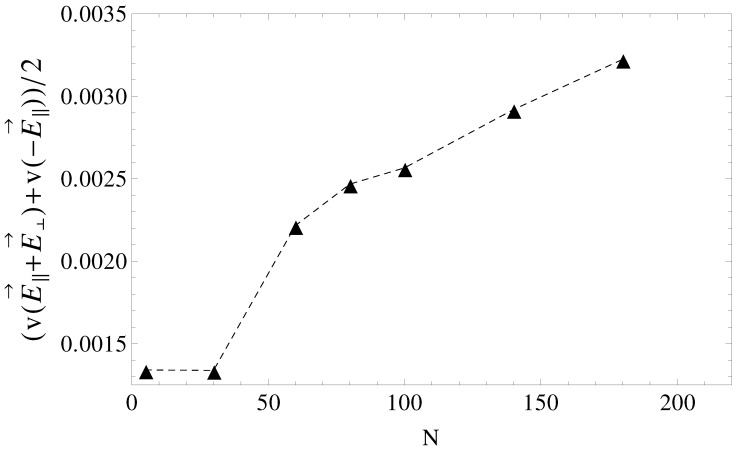
Average electrophoretic velocities as a function of chain length, *N*, for the case of pulsed transverse fields and pulsed longitudinal fields. During the first half of the pulse, the driving field is ${\vec{E}}_{∥}+{\vec{E}}_{⊥}$, while in the second half of the pulse, the field is switched to $-{\vec{E}}_{∥}$.

Considered separately, electrolyte friction and surface friction forces cannot predict the full correct order of velocities, $v_{T_{16}}<v_{P_{16}}<v_{P_{0}}<v_{T_{0}}$. Hydrodynamic friction alone cannot predict it either. The fluid velocity around the polyion (N=180) as a function of the radial distance *r* in smooth and wavy geometries was shown in Figure 3. It is readily understood that in geometry $T_{0}$, the fluid is partially trapped inside the polymeric domain (at r→0, $v_{fluid}^{z}$ approaches $v_{N=180}$), which means that from a hydrodynamic point of view, the transverse field makes the charged chains less porous to the fluid flow. This means that their hydrodynamic friction is reduced, because the hydrodynamic friction is proportional to the difference between the polyelectrolyte velocity and the local fluid velocity. In other words, the transversal field perturbs the free-draining property of the chains by moving the counterions away from the polymeric domain. We assume that this effect can explain the apparent increase in velocities in Figure 5 for geometries $T_{0}$ and $T_{2}$. On the other hand, as the number of undulations increases, the hydrodynamic resistance also increases, either in longitudinal or in transverse fields. It follows that electrolyte, hydrodynamic and surface friction effects are necessarily linked together and responsible for the observed velocities of the polyelectrolytes in confinement. If we restrict ourselves to smooth cylinder geometries, we can use the above understanding of the effect of transversal fields in order to improve separation resolution. For example, the applied field can be set to ${\vec{E}}_{∥}+{\vec{E}}_{⊥}$ for an arbitrary time interval and then switched to $-{\vec{E}}_{∥}$ for the same time interval. The scenario corresponds to pulsed longitudinal and transversal fields. The average velocities are shown in Figure 9 and are, in a first order approximation, equal to the mean of velocities, $v({\vec{E}}_{∥}+{\vec{E}}_{⊥})$ and $v(-{\vec{E}}_{∥})$, taken from Figure 5, geometries $P_{0}$ and $T_{0}$. It is clear that above N=30, separation can be achieved up to the longest chain length. Such calculations are a work in progress and will be detailed in a future publication.

## 4. Conclusions

We carried out molecular dynamics simulations of the electrophoresis of charged chains in cylinder geometries with smooth walls and undulations. The depth of the undulations with respect to the cylinder diameter has been kept constant, and only the wavelength has been varied. The driving electric field has been either constant and along the main axis of the cylinder geometry or a superposition of transversal and longitudinal fields. The transversal component offered, naturally, a way of controlling electrolyte friction, because it led to a separation of electro-osmotic flows of counterions and ions of salt. The electrolyte friction was found to be generally lower than in longitudinal fields only, because counterions were partially removed from the vicinity of the polyion, and thus, the counter electroosmotic flow was reduced. On the other hand, the removal of counterions led to a partial trapping of fluid monomers, which changed hydrodynamic friction, with the final result that in transversal fields, the polyelectrolytes were less permeable to the fluid flow. This effect resulted in a reduction in hydrodynamic friction. Surface undulations, however, slowed down the velocities, even in the presence of a transversal field. From a practical point of view, the present study shows that a series or combination of smooth channels with undulating surfaces, with longitudinal or transversal fields, may offer a way of accelerating or decelerating the motion of charged chains in confinement. The ultimate goal of such computer experiments, where one factor influences another, is to better understand the dynamics of the counterions and their role in electrophoresis.
